# Angiomyolipoma with epithelial cysts (AMLEC): a rare but distinct variant of angiomyolipoma

**DOI:** 10.1186/1746-1596-2-11

**Published:** 2007-03-21

**Authors:** Henry B Armah, Ming Yin, Uma NM Rao, Anil V Parwani

**Affiliations:** 1 Department of Pathology, University of Pittsburgh Medical Center, Pittsburgh, PA, USA

## Abstract

Angiomyolipoma with epithelial cysts (AMLEC) is a recently described distinct cystic variant of angiomyolipoma (AML). To date 15 cases of AMLEC have been reported in 2 case series. We report the 16^th ^case in a 39-year-old female. Her left kidney tumor was discovered incidentally. Partial nephrectomy was performed. Histologically, the tumor was composed of three components: 1) epithelial cysts lined by cuboidal to hobnail cells; 2) compact subepithelial mullerian-like AML stroma with admixed chronic inflammation; and 3) muscle-predominant AML with dysmorphic blood vessels exterior to the subepithelial stroma. Immunohistochemically, the subepithelial stroma stained most intensely with HMB-45 and Melan-A, whilst the muscle-predominant AML areas stained most intensely with smooth muscle actin and desmin. Estrogen receptor (ER), progesterone receptor (PR), and CD10 stained most intensely in the subepithelial stroma. The cyst lining was positive for pancytokeratin, but negative for HMB-45, Melan-A, ER, PR, and CD10. The patient is alive with no evidence of disease, 12 months postoperatively, and yearly follow-up CT scans are planned.

## Background

Angiomyolipomas (AMLs) are well-characterized triphasic tumors composed of varying amounts of vascular (thick-walled dysplastic or dysmorphic blood vessels), smooth muscle (spindled or epithelioid with clear cytoplasm) and mature adipose elements [1]. AML usually occurs in the kidney, but can occasionally involve the liver and retroperitoneum. AML comprise 2.0–6.4% of all renal tumors, however they represent one of the most common benign renal lesions [1]. AML can occur as an isolated renal lesion or as part of the tuberous sclerosis complex (TSC). Approximately 50% of patients with TSC develop AML, which tend to be bilateral and multifocal [2]. The triphasic nature of AML has led many in the past to consider these lesions as hamartomatous. However, recent detection of clonal genomic alterations [3-5] and rare case reports of malignancy in AMLs [1,6-8] favor their classification as neoplastic lesions. AMLs share morphologic and immunohistological features with perivascular epithelioid cell (PEC), and are considered to be among the growing family of tumors derived from these distinctive cells, also referred to as PEComas, and that includes clear cell ("sugar") tumors of the lung and pancreas, and lymphangioleiomyomatosis [9,10]. Although the diagnosis of AML is usually straightforward, some cases showing predominance of any one of the AML components may mimic a number of lesions and lead to an erroneous diagnosis of malignancy, including liposarcoma (fat-predominant AML), leiomyoma (muscle-predominant AML), renal cell carcinoma (epithelioid AML), and vascular malformations (paucicellular, vascular-predominant AML) [11]. The evolution of AML is classically benign, but malignant transformation has been rarely reported in 12 cases to date [1,6-8].

AMLs are typically solid lesions both radiologically and grossly [1], without cystic or epithelial components. Although entrapped non-cystic renal tubules have been described in AML, presentation as a cystic mass has been reported recently in only 15 cases in two case series [11,12]. This distinctive benign renal neoplasm has recently been recognized and termed angiomyolipoma with epithelial cysts (AMLEC) by Fine and colleagues [11] or cystic angiomyolipoma by Davis and colleagues [12]. These descriptive names for this entity are currently favored until its pathogenesis and relationships to other renal neoplasms are better understood. Therefore, AMLEC or cystic AML has to be considered in the differential diagnosis of adult cystic renal neoplasms, which includes cystic renal cell carcinomas, cystic nephroma (CN), and mixed epithelial and stromal tumor (MEST). The most distinctive immunohistochemical feature of AMLEC or cystic AML, absent in the above three tumors mentioned earlier is immunoreactivity with melanocytic markers (HMB45 and Melan-A) [11,12]. We report herein one more case of AMLEC or cystic AML in a 39-year-old female.

## Case presentation

A 39-year-old woman had a left kidney tumor incidentally discovered during CT scan as part of a diagnostic workup for colonic diverticulosis. She had no personal or family history of TSC, lymphangioleiomyomatosis, renal cyst, renal malignancy, or estrogen hormonal therapy. The CT scan revealed a 2.5-cm complex cystic mass in the upper pole of the left kidney with a 1-cm enhancing nodule in its wall, radiologically worrisome for cystic renal cell carcinoma. In view of this concern of malignancy, the patient elected to undergo laparoscopic left partial nephrectomy for definitive surgical treatment. The entire tumor was surgically resected with an excellent margin of 5-mm of normal parenchyma surrounding the entire cyst wall, and the tumor was confined to the kidney.

**Figure 1:**
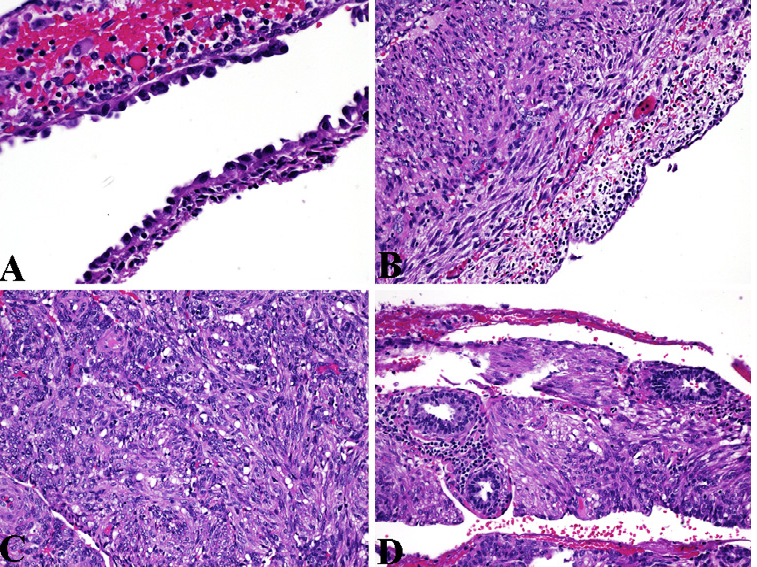
Histologic (H&E) findings of three components of AMLEC. (A) Epithelial cysts lined by cuboidal to hobnail cells. Original magnification X400. (B) Compact subepithelial "cambium-like" layer of cellular, mullerian-like AML stroma with prominent admixed chronic inflammation. Original magnification X200. (C) Muscle-predominant AML with associated dysmorphic blood vessels. Original magnification X200. (D) Non-cystic native tubules entrapped in muscle-predominant AML. Original magnification X200.

Grossly, the tumor was well demarcated and partially cystic, with the largest cyst measuring up to 1.1-cm. Sectioning of the tumor revealed part of the cyst wall contained a single 1-cm mural nodule with homogenous tan cut surface. The entire tumor was submitted for histological examination and revealed three components. The first component was cystic or multicystic spaces lined by epithelium, that ranged from flat to cuboidal to columnar. Whilst the cuboidal to columnar cells had unremarkable clear cytoplasm, the flat cells had abundant eosinophilic cytoplasm with nuclei that often protruded into the lumen, resulting in a hobnailed appearance (Figure 1A). The second component was a subepithelial "cambium-like" condensation of small stromal cells with indistinct cytoplasm immediately subjacent to the cyst epithelium. This subepithelial stroma showed prominent capillary vasculature (reminiscent of endometrial or mullerian-like stroma) and prominent lymphoplasmacytic infiltrate (Figure 1B). The third component was a thick exterior wall of plump smooth muscle cells with focally clear cytoplasm arranged in poorly formed fascicles, often appearing to emanate from irregular and tortuous blood vessels (Figure 1C). The third component was exterior to the subepithelial stroma and was typical of myomatous or muscle-predominant predominant AML. Additionally, non-cystic native renal tubules were observed entrapped within this exterior muscular wall (Figure 1D).

**Figure 2:**
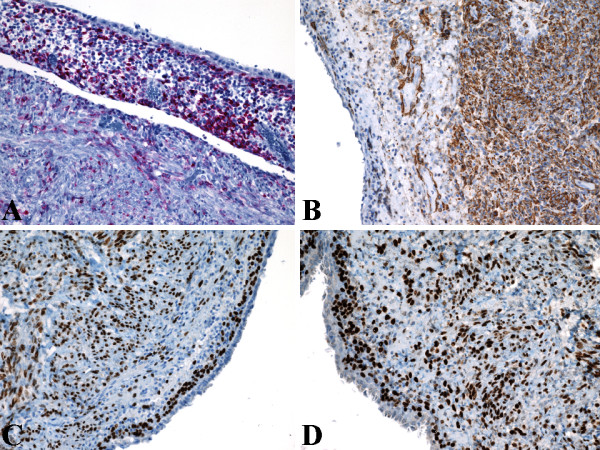
Immunohistochemical (IHC) findings of AMLEC. (A) Compact subepithelial stroma showed most intense HMB45 staining. Original magnification X200. (B) Muscle-predominant AML showed most intense smooth muscle actin staining. Original magnification X200. (C) Compact subepithelial stroma showed diffuse strong ER staining. Original magnification X200. (D) Compact subepithelial stroma showed diffuse strong PR staining. Original magnification X200.

**Figure 3:**
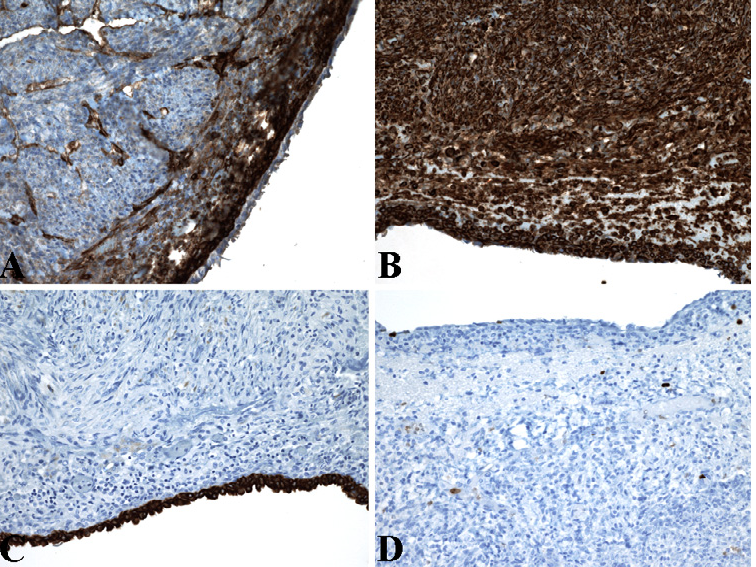
Immunohistochemical (IHC) findings of AMLEC. (A) Compact subepithelial stroma showed diffuse strong CD10 staining. Original magnification X200. (B) Diffuse strong vimentin staining in all 3 components equally. Original magnification X200. (C) Epithelium lining the cystic spaces showed strong intense pancytokeratin staining. Original magnification X200. (D) Ki67 staining showed low proliferative index of less than 1% of neoplastic cells. Original magnification X200.

Immunohistochemically, HMB45 (Figure 2A) and Melan-A labeling was patchy in the exterior muscle-predominant AML component, but were most intense and concentrated in the compact subepithelial cellular stroma. Conversely, smooth muscle actin (Figure 2B) and desmin labeling was most intense and concentrated in the exterior muscle-predominant AML component, but were patchy in the compact subepithelial cellular stroma. Similarly, the compact subepithelial cellular stroma showed strong and diffuse nuclear labeling for estrogen receptor (ER) (Figure 2C) and progesterone receptor (PR) (Figure 2D), along with strong and diffuse cytoplasmic labeling for CD10 (Figure 3A), but labeling for ER (Figure 2C), PR (Figure 2D), and CD10 (Figure 3A) were patchy in the exterior muscle-predominant AML component. However, vimentin (Figure 3B) showed strong and diffuse cytoplasmic labeling of all 3 components equally. The cyst lining was positive for epithelial markers (pancytokeratin [Figure 3C], AE1-AE3, and CK7), but negative for melanocytic (HMB-45 [Figure 2A] and Melan-A), muscular (smooth muscle actin [Figure 2B] and desmin), and hormonal (ER [Figure 2C] and PR [Figure 2D]) markers. The tumor showed low proliferative index with Ki67 labeling less than 1% of neoplastic cells (Figure 3D). Additionally, RCC marker antigen, inhibin, WT-1, c-kit (CD117), S-100 protein, and CK20 did not label any of the 3 components of the tumor (not shown). Except for patchy labeling of blood vessels, CD34 (endothelial markers) did not label any of the 3 components of the tumor (not shown). The patient herein presented is alive with no evidence of recurrence or metastatic disease, 12 months postoperatively, and follow-up with interval abdominal imaging studies is planned.

## Discussion

The renal tumor herein presented was histologically and immunophenotypically diagnostic of muscle-predominant AML containing prominent and grossly evident epithelial cysts. This phenotype is distinctly unusual, as AMLs are typically solid [1], and reminiscent of the recently described distinct cystic variant of AML that has been seperately designated as cystic AML or AMLEC [11,12]. It is well known that AMLs occur both in association with TSC and sporadically [1]. Bilateral or multiple AMLs have been considered presumptive evidence of, or diagnostic of, TSC [2]. The case herein presented had no personal or family history of TSC. From the 15 previously reported [11,12] and our case of AMLEC, only 1 out of these 16 cases of AMLEC was associated with TSC, suggesting that this rare variant of AML may not be related to TSC. Additionally, the female/male ratio is 10/6 for these 16 cases of AMLEC, indicating a slight female predominance for AMLEC. Therefore, unlike MEST which is considered estrogen hormone dependent because of its almost exclusive occurrence in females, AMLEC may be estrogen hormone independent. AMLs have been considered benign lesions and those found in the kidney are generally managed conservatively. Partial nephrectomy or angiographic embolization has been recommended for symptomatic lesions and lesions greater than 4-cm, and most asymptomatic lesions are followed with interval abdominal imaging [13]. However, 12 cases of metastatic AML have been reported [1,6-8]. Though the asymptomatic cystic renal tumor in the case herein presented was less than 4-cm in greatest dimension, definitive surgical treatment was pursued because of the presence of a radiologically enhancing nodule in the cyst wall, which was worrisome for malignancy.

AMLEC are readily distinct from most adult cystic renal lesions. The chief differential diagnostic consideration for AMLEC or cystic AML is mixed epithelial and stromal tumor (MEST), previously classified as cystic hamartomas of the renal pelvis, adult mesoblastic nephroma, or renal pelvic or cortical hamartomas [11,12]. Immunohistochemically, the stroma of both AMLEC and MEST labels for smooth muscle actin, desmin, ER, and PR [11,12]. However, the most distinctive immunohistochemical feature of AMLEC or cystic AML, absent in all the differential diagnostic considerations mentioned above, is immunostaining with melanocytic markers (HMB45 and Melan-A) [11,12], as noted in our case. This study further expands on the immunophenotype of this new histologic entity by reporting for the first time that AMLEC shows absence of immunoreactivity for WT-1, c-kit and CK20. The other important benign differential diagnostic consideration for AMLEC or cystic AML is cystic nephroma (CN). The main malignant differential diagnostic consideration for AMLEC or cystic AML is multilocular cystic renal cell carcinoma. Based on the results of immunohistochemical staining in the case herein presented, cystic and sarcomatous renal cell carcinoma (HMB45 negative), cystic nephroma (HMB45 negative), mixed epithelial and stromal tumor (HMB45 negative), leiomyosarcoma (HMB45 negative), and melanoma (HMB45 positive, S-100 protein positive) were excluded as differential diagnoses. The presence of HMB45 in PEC of AML has been widely recognized as a specific finding, however, that of c-kit (CD117) has not been as common [4,6,9]. According to recent reports, c-kit is also expressed in renal oncocytoma (71%), chromophobic renal cell carcinoma (85%) and even in PEC in classic AML [14,15]. The case we describe was not immunoreactive for c-kit.

The histogenesis of AMLEC or cystic AML is unclear. However, the histogenesis of the mullerian-like stroma in AMLEC has been postulated to be due to the embryological proximity between the urinary and genital systems [11]. These two systems share common origin from the urogenital ridge, and it has been postulated that disturbances during a critical period in development may lead to crossover of epithelium or mesenchymal elements between the two systems, predisposing to neoplasms that combine these features [11]. The strong HMB-45 positivity of the "cambium-like" layer of compact subepithelial cells in AMLEC supports the concept that they are a variant of AML, although their morphology is distinctly different from the exterior muscle-predominant AML wall. The mullerian histomorphology and peculiar immunohistochemical profile (HMB45+, Melan-A+, ER+, PR+, and CD10+) of the compact subepithelial cells suggests both mullerian and melanocytic differentiation of PECs in AMLEC, a rare variant of AML [11]. This observation of dual differentiation is not unprecedented in AML, since the smooth muscle cells of AMLs are known to have both melanocytic and muscular features [1,4,6,9]. The minimal immunoreactivity for muscle markers in this subepithelial zone suggests that these cells have lost some of their muscular phenotype while developing a mullerian phenotype. Apart from the fact that the presence of epithelium is extremely uncommon in AML and has been reported previously in only 15 cases [11,12], the nature of the epithelium within AMLEC is also controversial. Davis and colleagues [12] favored the view that the epithelial component of AMLEC represented true epithelial differentiation by the AML, whilst Fine and colleagues [11] favored the view that it mainly represented dilated entrapped native renal collecting duct epithelium. Both views are plausible.

## Conclusion

AMLEC should be routinely included in the differential diagnostic considerations for adult cystic renal neoplasms, which includes cystic renal cell carcinomas, cystic nephroma (CN), and mixed epithelial and stromal tumor (MEST). Although, AMLEC may be confused with MEST, the most distinctive feature is the fact that AMLEC is immunoreactive to melanocytic markers (HMB45 and Melan-A).

## Competing interests

The author(s) declare that they have no competing interests.

## Authors' contributions

**HBA **participated in the histopathological evaluation, performed the literature review, acquired photomicrographs and drafted the manuscript. **MY **participated in the grossing of the tumor, participated in the histopathological evaluation and contributed suggestions for drafting the manuscript. **UNMR **reviewed the histopathological diagnosis and critically revised the manuscript for important intellectual content. **AVP **conceived and designed the study, gave the histopathological diagnosis and revised the manuscript for important intellectual content. All authors read and approved the final manuscript.
